# Glucose metabolism, lipid metabolism, and body composition of pregnant women personalized nutritional management based on pre-pregnancy BMI and percent body fat

**DOI:** 10.3389/fnut.2026.1780921

**Published:** 2026-05-29

**Authors:** Zhen Ding, Lingmei Zhou, Kemei Jin, Zhanfeng Li, Yinfei Huang, Xia Cen, Jinghui Zou

**Affiliations:** 1 Clincal Nutrition Department, The Affiliated Li Huili Hospital of Ningbo University, Zhejiang, China; 2 Obstetrics Department, The Affiliated Li Huili Hospital of Ningbo University, Zhejiang, China

**Keywords:** body composition, body fat percentage, gestational diabetes mellitus, personalized nutrition management, pre-pregnancy body mass index

## Abstract

**Purpose:**

To describe glucose metabolism, lipid metabolism, and body composition of pregnant women personalized nutritional management based on pre-pregnancy body mass index (BMI) and body fat percentage (PBF).

**Methods:**

Pregnant women who registered for prenatal examinations at Li Huili Hospital, Ningbo Medical Center, between January 2021 and January 2022 were selected. They were randomly assigned to a control group (47 cases) and a management group (57 cases). According to the Oral Glucose Tolerance Test diagnostic criteria, the management group was further divided into a gestational diabetes mellitus (GDM) group (28 cases) and a non-GDM group (29 cases). The control group received routine prenatal check-ups and follow-up management, including weight, uterine height, and abdominal circumference monitoring. The management group received personalized nutritional management guided by dynamic monitoring of body composition.

**Results:**

(1) Compared with the control group, women in the GDM management group had higher gestational age, pre-pregnancy BMI, and gestational weight gain (*p* < 0.01, 0.006, and 0.03, respectively). Their spontaneous delivery rate (35.71%) was significantly lower than that of the control group (80.85%; *p* = 0.002). (2) During the second trimester of pregnancy, in both the control and non-GDM groups, fasting, 1-h, and 2-h blood glucose levels were lower than those in the GDM group (*p* < 0.05). However, in the later stage of pregnancy, no significant differences were observed in blood glucose among the three groups. (3) Weight (*p* = 0.02), body fat mass (BMF), PBF, and visceral fat area (*p* < 0.01) were higher in the GDM group than in the non-GDM group.

**Conclusion:**

(1) Personalized nutritional management may be effective for administrating gestational weight gain (GWG). It may also help control blood glucose during late pregnancy for women with GDM. (2) Increased early pregnancy weight, BMF, and PBF may be associated with a risk of developing GDM. (3) Body composition testing during early pregnancy could help identify individuals at high risk for GDM, for whom personalized nutritional management could be implemented early in their pregnancy.

## Introduction

Overweight, obesity, and related chronic diseases have become major global public health challenges across age and sex groups. Excess maternal weight increases the risk of spontaneous abortion, congenital anomalies, insulin resistance, lipid and amino acid metabolic disorders, increased premature/stillbirth, birth injuries, and cesarean section. Postpartum weight retention also contributes to “developmental metabolic syndrome” in the offspring (1). The Guidelines for Medical Nutritional Treatment of Overweight/Obesity in China (2021) highlight that lifestyle interventions, especially dietary adjustments, in overweight or obese pregnant women can improve weight gain and promote maternal-infant health (2). Although body mass index (BMI) is a simple screening tool, it cannot reflect fat mass or fat distribution (3), both of which independently influence the risk of gestational diabetes mellitus (GDM). Therefore, we aimed to evaluate the necessity of personalized nutritional management based on pre-pregnancy BMI and early-pregnancy body fat percentage (PBF).

## Materials and methods

### Participants

Pregnant women who attended prenatal examinations at Li Huili Hospital, Ningbo Medical Center, between January 2021 and January 2022 were enrolled. Inclusion criteria: ① Age 18–40 years; ② Singleton pregnancy; ③ Willingness to undergo full-course nutrition management and routine prenatal check-ups. Exclusion criteria: ① Pre-existing diabetes mellitus, or pregnancy complicated by hyperthyroidism / hypothyroidism; ② Patients with severe heart, liver, or kidney disease; ③ Those taking hormonal drugs during pregnancy; ④ Refusal to participate in this study.

### Methods

All enrolled pregnant women were randomly assigned to either the management or the control group using a random number table. The control group underwent regular prenatal check-ups and follow-up management, including monitoring of weight, uterine height, and abdominal circumference. The management group received personalized nutrition management based on dynamic monitoring of body composition. The specific plan included: ① Dynamic monitoring of body composition: body composition detection was performed in early, mid, and late pregnancy (Inbody 770); ② Estimation of total daily energy intake according to pre-pregnancy BMI and early-pregnancy PBF (total daily energy = ideal weight × energy coefficient; ideal weight = height (cm)-105; coefficient 25–30 kcal/kg/d). Energy intake increased by 250 kcal/day in the second trimester and 400 kcal/day in the third trimester, with a minimum of 1,600 kcal/day in the first trimester and 1,800–2200 kcal/day thereafter; ③ Three-day dietary records with adjustments by a nutritionist; (4) Continuous monitoring of weight gain, PBF, and skeletal muscle mass, with tailored advice on aerobic and resistance exercise (4, 5).

### Observation indicators

① The following basic information for each participant was collected through the women's health care system and the inpatient medical record collection system: age, parity, pre-pregnancy BMI, gestational age, gestational weight gain, delivery mode, newborn weight, hospitalization days, and macrosomia (≥ 4,000 g). ② Laboratory indicators: fasting blood glucose and lipid levels in early and late pregnancy as well as Oral Glucose Tolerance Test (OGTT) results at 24–28 weeks. According to the 2022 guidelines for the diagnosis and treatment of hyperglycemia in pregnancy, a 75 g OGTT was used as the diagnostic indicator of GDM. The blood glucose thresholds at 1 h and 2 h after fasting and oral glucose were 5.1, 10.0, and 8.5 mmol/L, respectively. GDM was diagnosed at any time point when blood glucose levels reached or exceeded the above criteria (6). Body composition indices measured in the management group included body weight, skeletal muscle mass, body fat mass, PBF, and visceral fat area (VFA) from early to late trimester.

### Statistical analyses

SPSS 23.0 statistical software was utilized to process and analyze the data. Paired *t*-test was employed for normally distributed continuous data and Chi-square test was used for categorical data when comparing results between the control and the management groups. Kruskcal–Wallis test were applied to compare data among the control group, GDM group, and non-GDM groups. They were also used to assess body composition in the management group during the first, second, and third trimesters of pregnancy. Then, pairwise comparisons were conducted for all positive results. All statistical analyses were performed using two-tailed tests, with a significance level of *p* < 0.05.

## Results

### Patient characteristics

A total of 47 pregnant women in the control group and 57 in the management group were included. According to the OGTT diagnostic criteria, women in the management group were further classified into a non-GDM group (*n* = 29) and a GDM group (*n* = 28). The general clinical characteristics of the three groups are presented in Table 1. Compared with the control group, the age and pre-pregnancy BMI of participants in the GDM group were higher (*p* < 0.01, *p* = 0.006, respectively), while their weight gain during pregnancy was lower than that in the control group (*p* = 0.030). The natural delivery rate was also significantly lower in the GDM group (35.71%) than in the control group (80.85%). No significant differences were found in the remaining variables.

**Table 1:** Comparison of general clinical data between the control group and the management group of pregnant women.

Clinical data		Control group (*N* = 47)	Management group (*N* = 57)		Test value	*P*-value
			Non GDM group (*n* = 29)	GDM group (*n* = 28)		
Age (years)		28.45 ± 3.76	30.65 ± 4.07	33.25 ± 2.79^1^	14.290	< 0.01^*^
Week of gestation		39.26 ± 0.94	39.17 ± 0.97	39.10 ± 0.90	0.208	0.812
BMI before pregnancy (kg/m^2^)		20.36 ± 3.11	22.21 ± 2.60	22.81 ± 2.78^1^	5.504	**0.006** ^*^
Gestational weight gain (kg)		14.74 ± 4.65	12.86 ± 4.42	11.32 ± 5.25^1^	3.673	**0.030** ^*^
Parity	1	31 (65.96%)	25 (86.21%)	21 (75.00%)	2.908	0.234
	2	16 (34.04%)	4 (13.79%)	7 (25.00%)		
Mode of delivery	Natural delivery	38 (80.85%)	21 (72.41%)	10 (35.71%)^1^	12.101	0.002^*^
	Cesarean section	9 (19.15%)	8 (27.59%)	18 (64.29%)		
Macrosomia	No	44 (93.62%)	28 (96.55%)	26 (92.86%)	0.854	0.652
	Yes	3 (6.38%)	1 (3.45%)	2 (7.69%)		
Newborn weight (kg)		3.41 ± 0.37	3.28 ± 0.24	3.39 ± 0.33	0.794	0.456
Hospitalization days (days)		5.53 ± 1.59	6.00 ± 2.12	6.25 ± 2.12	1.367	0.267

^1^Compared with the control group, *p* < 0.05. ^*^The difference between control and management group was siginificant.

### Comparison of glucose and lipid indexes

The blood glucose, blood lipid indices, and triglyceride-glucose (TyG) index of the three groups during early, mid-, and late pregnancy are presented in Table 2. Fasting 1-h and 2-h blood glucose levels in the non-GDM group and the control group were lower than those in the GDM group. There was no difference in these indices among the three groups during early- and late-pregnancy.

**Table 2:** Comparison of glucose and lipid indexes in pregnant women in control group and management group.

Glucose and lipid indexes	Control group (*N* = 47)	Management group (*N* = 57)		Test value	*P*-value
		Non GDM group (*n* = 29)	GDM group (*n* = 28)		
Early-pregnancy					
FPG	4.74 ± 0.50	4.51 ± 0.43	4.90 ± 0.45	−0.184	0.854
TyG	4.58 ± 0.23	4.68 ± 0.37	4.67 ± 0.43	−0.6.22	0.546
Mid-pregnancy					
FPG	4.41 ± 0.29^1^	4.46 ± 0.29^2^	4.87 ± 0.61	−2.806	0.007^*^
1hPBG	6.96 ± 1.36^1^	7.33 ± 1.40^2^	9.91 ± 1.38	−5.554	< 0.01^*^
s2hPBG	6.12 ± 0.99^1^	6.48 ± 1.30^2^	8.14 ± 1.38	−4.309	< 0.01^*^
Late-pregnancy					
FPG	4.09 ± 0.57	4.30 ± 0.71	4.39 ± 0.73	−1.786	0.078
TG	3.46 ± 1.41	2.92 ± 1.05	4.07 ± 2.57	−0.32	0.749
TC	6.34 ± 1.08	5.96 ± 0.94	5.86 ± 1.30	1.727	0.088
HDL-C	1.99 ± 0.49	1.72 ± 0.29	1.93 ± 0.61	1.293	0.200
LDL-C	3.43 ± 0.46	3.50 ± 0.74	3.16 ± 0.89	0.740	0.461
TyG	4.97 ± 0.20	4.96 ± 0.21	4.80 ± 1.16	0.798	0.427

^1, 2^Compared with the GDM group, both 1 and 2 showed statistically significant differences (*p* < 0.05). ^*^The difference between control and management group was siginificant.

### Comparison of body composition characteristics

**Figure 1:**
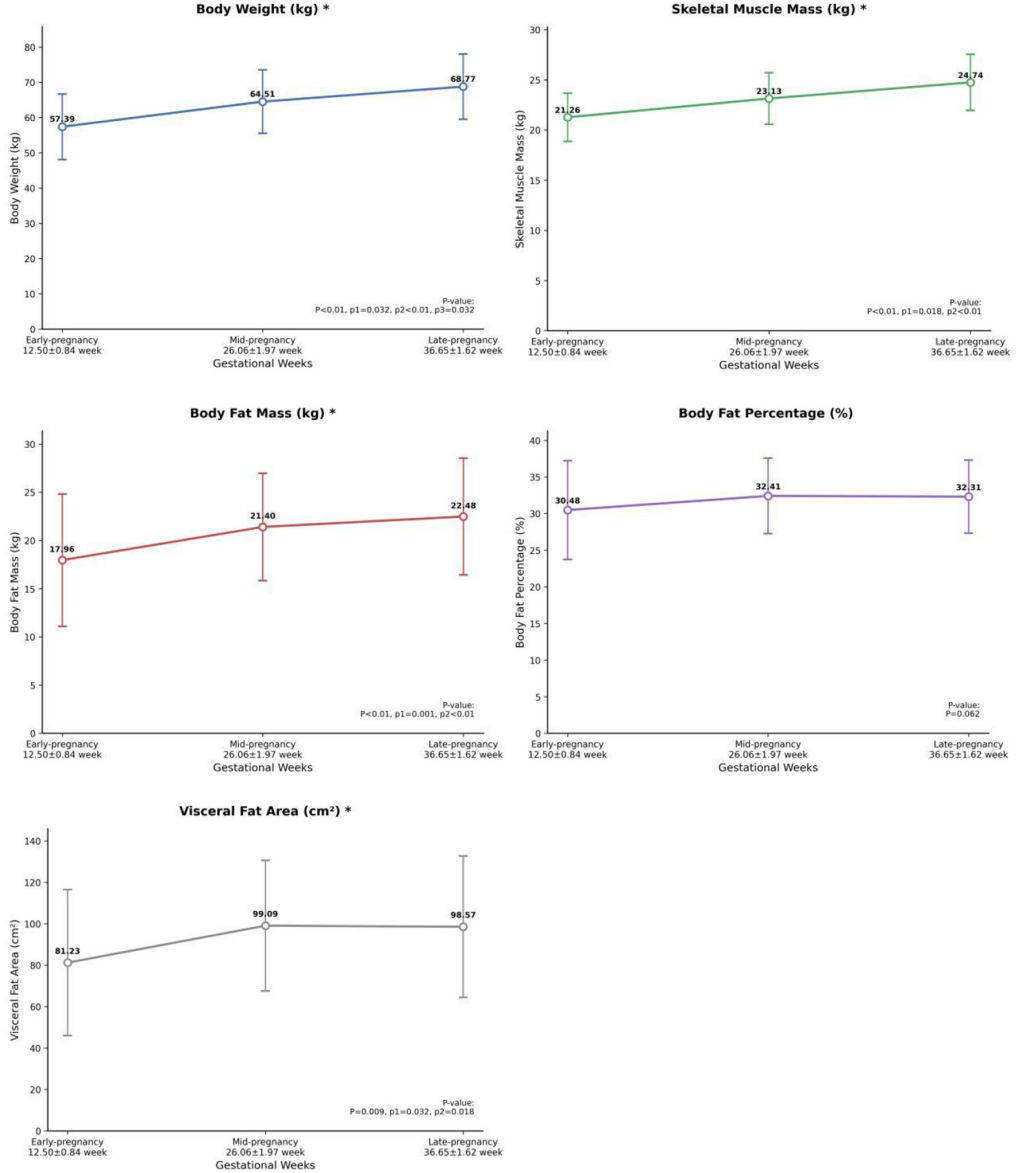
Fluctuations of body composition in pregnant women in the management group. *Fluctuations showed statistically significant differences (*p* < 0.05); *P*: comparison among early, mid, and late pregnancy groups; p1: comparison between mid and early pregnancy; p2: comparison between late and early pregnancy; p3: comparison between late and mid pregnancy.

The weight of all participants in the management group gradually increased from the first trimester to the third trimester (*p* < 0.01). There were no differences in skeletal muscle, body fat mass, or VFA between the second and third trimesters; however, the three indexes in the second and third trimesters were higher than the first trimester. No significant difference in PBF was observed during pregnancy (*p* = 0.062; Figure 1).

The comparison between the GDM group and the non-GDM group showed that, in early-pregnancy, the weight (*p* = 0.02), body fat mass, PBF, and VFA (all *p* < 0.01) of participants in the GDM group were higher than those in the non-GDM group. No significant differences in body composition were observed between the groups during mid- and late- pregnancy (Table 3).

**Table 3:** Comparisons of body composition index between GDM group and the non-GDM group.

Pregnancy Stage	Gestational weeks (week)		Weight (kg)		Skeletal muscle (kg)	
	Non-GDM group (*n* = 29)	GDM group (*n* = 28)	Non-GDM group (*n* = 29)	GDM group (*n* = 28)	Non-GDM group (*n* = 29)	GDM group (*n* = 28)
Early-pregnancy	12.06 ± 1.91	13.36 ± 0.81	55.57 ± 8.40	65.55 ± 9.76	21.08 ± 2.57	22.10 ± 1.08
	*t* = 1.89, *p* = 0.08		***t*** **=** –**2.55*****, p*** **=** 0.02^*^		*t* = −0.94, *p* = 0.35	
Mid- pregnancy	26.66 ± 1.98	25.57 ± 1.87	63.75 ± 9.97	65.13 ± 8.30	23.04 ± 2.48	23.21 ± 2.69
	*t* = 1.73, *p* = 0.09		*t* = −0.46, *p* = 0.65		*t* = −0.20, *p* = 0.84	
Late-pregnancy	37.12 ± 1.11	36.22 ± 1.92	70.78 ± 9.62	66.92 ± 7.72	25.66 ± 2.87	24.02 ± 2.59
	*t* = 1.36, *p* = 0.19		*t* = 1.01, *p* = 0.33		*t* = 1.49, *p* = 0.15	
	Body fat mass (kg)		Body fat percentage (%)		Visceral fat area (cm^2^)	
	Non-GDM group (*n* = 29)	GDM group (*n* = 28)	Non-GDM group (*n* = 29)	GDM group (*n* = 28)	Non-GDM group (*n* = 29)	GDM group (*n* = 28)
Early-pregnancy	16.56 ± 5.50	24.73 ± 9.15	29.17 ± 5.86	36.83 ± 7.62	74.31 ± 30.55	114.67 ± 40.11
	***t*** **=** –**2.94**, ***p*** **=** 0.01^*^		***t*** **=** –**2.78*****, p*** **=** 0.01^*^		***t*** **=** –**2.80*****, p*** **=** 0.01^*^	
Mid- pregnancy	21.18 ± 6.38	21.56 ± 5.01	32.52 ± 5.14	32.32 ± 5.27	96.04 ± 35.44	101.68 ± 28.47
	*t* = −0.21, *p* = 0.83		*t* = 0.12, *p* = 0.91		*t* = −0.54, *p* = 0.60	
Late-pregnancy	22.83 ± 6.67	22.20 ± 5.69	32.47 ± 5.02	32.18 ± 5.13	98.17 ± 40.84	98.89 ± 29.41
	*t* = 0.28, *p* = 0.78		*t* = 0.16, *p* = 0.88		*t* = −0.05, *p* = 0.96	

^*^comparison between non-GDM and GDM groups of pregnant women. *p* < 0.05.

## Discussion

Age, pre-pregnancy BMI, and weight gain during pregnancy are important risk factors for GDM (7). In our results, participants in the GDM group had higher gestational age and pre-pregnancy BMI than those in the control group, whereas their weight gain during pregnancy was lower. Previous studies have shown that excessive weight gain in early pregnancy increases the risk of GDM in women with normal pre-pregnancy BMI (8, 9). The occurrence of adverse pregnancy outcomes in pregnant women who were overweight or obese before pregnancy was not affected by weight gain during pregnancy, and their pre-pregnancy BMI was more strongly correlated with pregnancy outcomes (10). The BMI of pregnant women included in this study was normal before pregnancy, but the weight gain of participants in the GDM group was lower than that of the control group, likely due to personalized nutrition management. A meta-analysis has reported that dietary and exercise interventions aimed at reducing weight gain during pregnancy could reduce the incidence of GDM (11). In addition to weight gain during pregnancy, the combined triglyceride-glucose (TyG) index during pregnancy predicts adverse pregnancy outcomes, including macrosomia (12). TyG, an index that can reflect the degree of insulin resistance, has a certain predictive value for insulin sensitivity and can be used to predict GDM (13). Yanbei et al. (14) reported that the cut-off value of TyG to predict GDM in the first trimester in the Chinese population was 8.10 mmol/L. In this study, no difference in early blood glucose and TyG levels was found between pregnant women in the management and control groups, and TyG did not reach the cut-off value of previous studies. With personalized nutritional management, late-pregnancy blood glucose levels in the GDM group were not significantly different from those of the other groups.

The implementation of personalized nutritional management, which include tailored dietary and exercise interventions, was based on dynamic monitoring of body composition. Therefore, in addition to pre-pregnancy BMI and TyG levels during pregnancy, attention should also be paid to muscle, fat, and other body composition. Bioelectrical impedance analysis (BIA) is quick, safe during pregnancy, and user-friendly. However, BIA cannot separate maternal tissues from fetal tissues (15). Thus, the fluctuations of body composition for all pregnant women in the management group were tested, from the early to the late stage of pregnancy. The difference in body composition was mainly reflected as an increase in body fat mass, skeletal muscle, and VFA at the beginning of the second trimester. There was no significant difference between the second and third trimesters. The weight, body fat mass, PBF, and VFA of participants in the GDM group were significantly higher than those in the non-GDM group; however, there were no significant differences between the two groups in the second and third trimesters. A higher body fat mass in early pregnancy indicates more adipose tissue, which can cause a large secretion of free fatty acids and induce an inflammatory response, leading to increased secretion of inflammatory factors, which in turn affects insulin sensitivity and induces insulin resistance, thus increasing the risk of GDM (16). A study by Liu et al., (17) which included 1,318 pregnant women, showed that a higher percentage of body fat increased the risk of GDM, whereas a higher percentage of skeletal muscle was protective. Therefore, pregnant women should be provided guidance on how to increase resistance exercise and protein supplementation, increase muscle weight, and reduce PBF, all of which may play a protective role in GDM. Cohort studies have shown that each standard deviation increase in early pregnancy weight gain—within Institute of Medicine recommendations—raises GDM risk by 23%, while mid-pregnancy weight gain is not associated with GDM (18). Some scholars speculate that the reason may be that, compared to middle and late pregnancy, most of the weight gained in early pregnancy is adipose tissue (19), which has a greater impact on subsequent insulin resistance and impaired glucose tolerance (18). In conclusion, we recommend that body composition testing should be included into routine prenatal examinations, with particular attention to the dynamic fluctuation of body fat mass and lean body mass during the first and second trimesters. Thus, personalized nutritional interventions for pregnant women at high risk of GDM can be implemented as early as possible.

Our study showed that gestational weight gain in pregnant women in the management group was lower than that in the control group, and there was no significant difference in late-pregnancy blood glucose levels among GDM patients between the two groups. Although these outcomes may be attributed to our personalized nutritional management, this study was limited by its small sample size and lack of body composition data of the control group. Therefore, it is not possible to determine the effect of our personalized nutritional intervention on body composition data during the mid-to-late stages. Additionally, we could only identify the risk factors for GDM through retrospective analysis and were unable to establish causal inferences. Therefore, we are currently conducting a larger study with larger subgroups of high-risk pregnant women cohorts, with higher pre-pregnancy BMI and PBF. Precision nutrition management will be implemented and pregnancy outcomes will be compared. Meanwhile, additional risk factors related to microecology, metabolomics, and phenomics are worth exploring, as they can provide new insights and standardized approaches for preventing metabolic diseases, including GDM and maternal and childhood obesity.

## Data Availability

The original contributions presented in the study are included in the article/supplementary material, further inquiries can be directed to the corresponding author.
